# The Developmental Intestinal Regulator ELT-2 Controls p38-Dependent Immune Responses in Adult *C*. *elegans*

**DOI:** 10.1371/journal.pgen.1005265

**Published:** 2015-05-27

**Authors:** Dena H. S. Block, Kwame Twumasi-Boateng, Hae Sung Kang, Jolie A. Carlisle, Alexandru Hanganu, Ty Yu-Jen Lai, Michael Shapira

**Affiliations:** 1 Department of Integrative Biology, University of California, Berkeley, Berkeley, California, United States of America; 2 Graduate Group in Microbiology, University of California Berkeley, Berkeley, California, United States of America; Genentech, UNITED STATES

## Abstract

GATA transcription factors play critical roles in cellular differentiation and development. However, their roles in mature tissues are less understood. In *C*. *elegans* larvae, the transcription factor ELT-2 regulates terminal differentiation of the intestine. It is also expressed in the adult intestine, where it was suggested to maintain intestinal structure and function, and where it was additionally shown to contribute to infection resistance. To study the function of *elt-2* in adults we characterized *elt-2*-dependent gene expression following its knock-down specifically in adults. Microarray analysis identified two ELT-2-regulated gene subsets: one, enriched for hydrolytic enzymes, pointed at regulation of constitutive digestive functions as a dominant role of adult *elt-2*; the second was enriched for immune genes that are induced in response to *Pseudomonas aeruginosa* infection. Focusing on the latter, we used genetic analyses coupled to survival assays and quantitative RT-PCR to interrogate the mechanism(s) through which *elt-2* contributes to immunity. We show that *elt-2* controls p38-dependent gene induction, cooperating with two p38-activated transcription factors, ATF-7 and SKN-1. This demonstrates a mechanism through which the constitutively nuclear *elt-2* can impact induced responses, and play a dominant role in *C*. *elegans* immunity.

## Author Summary

*C*. *elegans* provides a tractable genetic model to study the regulation of the evolutionarily conserved innate immune system. One of the central signaling modules of innate immunity in all organisms is the p38 pathway, which has been studied extensively in *C*. *elegans*. Such studies identified the transcription factors ATF-7 and SKN-1 as proteins mediating downstream effects of the p38 pathway on immune and oxidative stress gene expression. Previous studies in *C*. *elegans* also identified ELT-2, a conserved transcription factor important for intestinal development, as a major regulator of immune responses in the adult worm. The current study aimed to characterize the interactions between these two immune regulatory modules. Microarray gene expression analysis in animals with disrupted *elt-2* expression revealed two gene subsets that were regulated by *elt-2*: one that included constitutively regulated genes, and was mostly comprised of digestive enzyme genes, and a second that included genes induced by infection with *Pseudomonas aeruginosa*. Both subsets were enriched for p38 targets. Genetic analyses and gene expression measurements of *elt-2*-regulated genes demonstrated that *elt-2* cooperates with the p38 pathway and its downstream mediators. These results suggest that ELT-2 functions as a tissue-specific master regulator controlling the contribution of the p38 MAPK pathway to innate immune responses.

## Introduction

Induction of local innate immune responses is the first reaction to an invading pathogen, and includes increased expression of antimicrobial effector peptides/proteins, as well as immune modulators. Regulation of these responses depends on signaling modules that are similar in their principles of action from plants to animals, suggesting convergent evolution [1]. Within the animal kingdom these signaling modules often use similar proteins, such as pattern recognition receptors, their downstream signaling cascades, and MAP kinase signaling pathways [2,3]. This conservation warrants the study of innate immune mechanisms in well-characterized invertebrate model organisms, such as *Drosophila melanogaster* and *Caenorhabditis elegans*, to better understand their vertebrate counterparts.

Studies of *C*. *elegans* immunity have repeatedly converged on the p38 MAPK pathway as a pivotal module in orchestrating immune responses, very similar to its roles in vertebrate innate immune responses [4–7]. The core components of the *C*. *elegans* p38 pathway include the NSY-1 MAP3K, the SEK-1 MAP2K, and the PMK-1 MAPK. TIR-1/SARM was shown to serve as an upstream activator during infection [8,9], and VHP-1/DUSP8, as a negative regulator [10]. Downstream to the p38 pathway, several transcription factors have been shown to mediate effects on gene expression: ATF-7, an ATF-2 ortholog, was shown to regulate immune gene expression in the intestine [11]; DAF-19/RFX, was shown to cooperate with ATF-7 in regulating genes involved in neuronal serotonin synthesis, but was also found to contribute to expression of intestinal immune genes [12]; SKN-1/Nrf, better known for regulating oxidative stress responses, was further found to contribute to resistance against bacterial pathogens [13–15]. In addition, ELT-3 was identified as a regulator of epidermal anti-fungal responses, a subset of which was also regulated by the p38 pathway [16].

ELT-3 is one of two *C*. *elegans* transcription factors of the GATA family with roles in epithelial development and differentiation, and additional roles in regulating immune responses. ELT-3 is important for epidermal differentiation and epidermis-specific gene expression [17]. The second GATA protein is ELT-2, which is important for terminal development of the intestine and for intestine-specific gene expression [18,19]. Whereas ELT-2 was proposed to be the predominant regulator of all intestinal gene expression, experiments supporting this were performed only in embryos or L1 larvae, leaving the extent of its roles in the adult intestine unresolved [20,21]. We, and others, have shown that ELT-2 regulated specific anti-bacterial responses in the adult intestine [22–24]. Similar roles, both in endodermal development, as well as in adult immune regulation and protection, were described for the *Drosophila* GATA protein *Serpent* and for the vertebrate GATA6 [22,25].

Vertebrate GATA transcription factors comprise two homology groups: GATA1-3 are regulators of lymphocyte terminal differentiation and cytokine expression; GATA4-6 are regulators of mesodermal and endodermal differentiation (in the heart, liver, lung, and pancreas), and are considered the orthologs of *elt-2* [26,27]. In the adult endoderm, GATA4 and GATA6 were also shown to play key roles in the regulation of stress responses [28,29]. Importantly, MAPK signaling, including signals from the p38 pathway, regulates the activity of GATA4 during stress responses [30]. Thus, it is possible that ELT-2 is similarly regulated during infection.

To better understand the roles of ELT-2 in the adult intestine, particularly its involvement in immune gene regulation, we characterized gene expression following *elt-2* knock-down specifically in adults. This identified two gene subsets: one that was constitutively regulated by ELT-2 and included genes involved in digestive degradation of macromolecules; and a second, which was induced in response to infection, and included genes previously implicated in protection from pathogens. Members of the latter demonstrated co-regulation by ELT-2 and the p38 pathway. Subsequent genetic analyses identified genetic interactions between *elt-2* and the p38 transcriptional mediator genes *atf-7* and *skn-1* in regulating *C*. *elegans* innate immune responses. Our results suggest a dominant role for *elt-2* in the regulation of digestive and metabolic functions of the intestine, and the role of a master regulator for p38-dependent immune responses, cooperating with activated transcription factors to control induced responses.

## Results

### The constitutive and inducible *elt-2* regulon

To identify genes regulated by *elt-2*, we compared gene expression profiles in animals fed with elt-2 RNAi during the first two days of adulthood (RNAi-ad) to those in control-treated animals, either following a twelve hour infection with *Pseudomonas aeruginosa*, or exposure to non-pathogenic *E*. *coli* (Raw data can be downloaded from GEO, accession no. GSE63846). Adult *elt-2* knock-down has been shown to cause a marked decrease in ELT-2 protein levels persisting up to three days after worms were removed from RNAi plates [22]. Successful knock-down is also discernible by eye, as animals present a modest ‘clear’ phenotype, potentially due to reduced fat storage (S1A Fig). Previous work found *elt-2(RNAi-ad)* animals to be more susceptible to infection, but to have a normal lifespan on dead *E*. *coli*, suggesting that effects of post-developmental *elt-2* knock-down are largely immune-specific [22].

**Fig 1:**
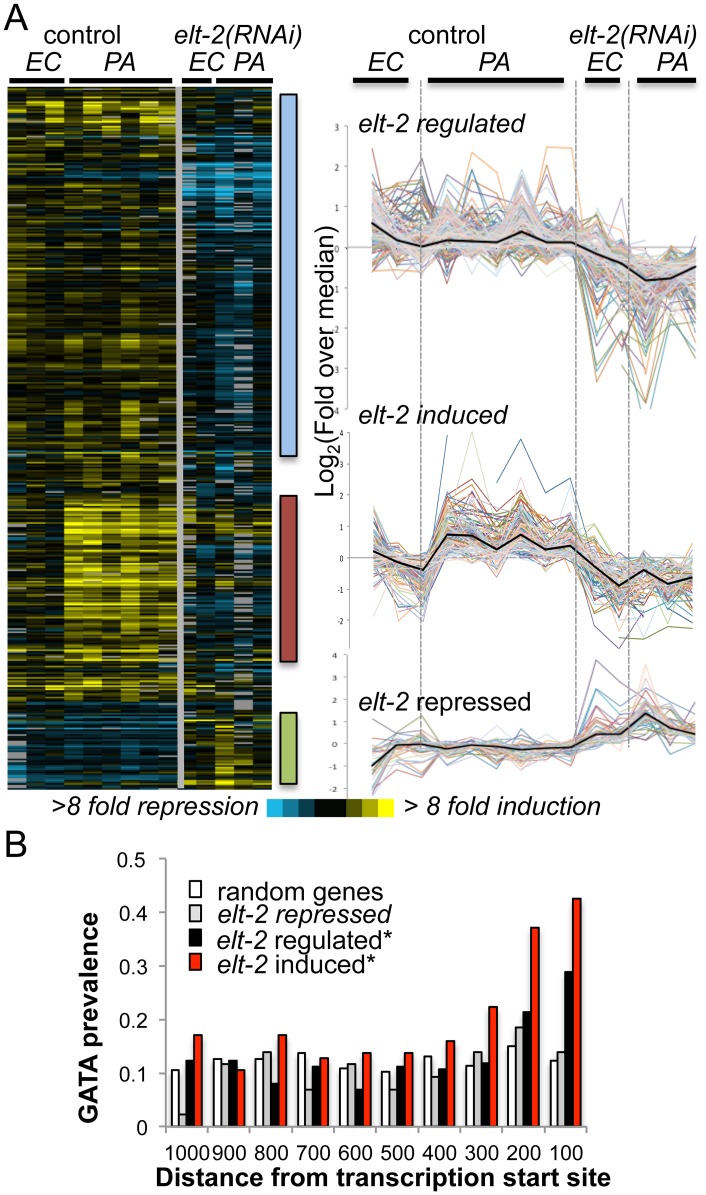
*elt-2* regulated genes. *A*. *microarray analysis*. Gene expression profiles for 426 transcripts differentially expressed between *elt-2(RNAi)* and control-treated wildtype worms, when exposed to *P*. *aeruginosa* (PA, 12 hours), compared to *E*. *coli* (*EC*). Left, heatmap of raw values (log_2_(fold change over reference RNA)), with bars highlighting clusters of interest; right, curves depicting median-centered expression profiles; black curves represent the median. *B*. *GATA motif distribution*. Measured for the consensus TGATAA in 1000bp upstream sequences of genes of the designated subsets; shown as #motifs/gene/100bp; asterisks mark significant deviations from random distribution (p<10^–8^, χ^2^).

Microarray analysis identified 429 transcripts, corresponding to 420 genes, which were differentially expressed in *elt-2(RNAi)* animals compared to control-treated animals (Fig 1A). Prominent clusters of co-regulated genes included a cluster of 187 genes with reduced expression following *elt-2* knock-down (‘*elt-2*-regulated’), suggesting contribution of *elt-2* to constitutive expression (Fig 1A and S2 Table); a cluster of 96 genes, that were also suppressed following *elt-2* knock-down, and additionally failed to be induced by infection in *elt-2(RNAi)* animals (‘*elt-2*-induced’); and a cluster of 43 genes showing elevated expression following *elt-2* knock-down, suggesting repression by the transcription factor (‘*elt-2*-repressed’). qRT-PCR verified *elt-2* regulation for three selected ‘*elt-2*-regulated’, and seven ‘*elt-2*-induced’ genes (S2A and S2B Fig). Additional measurements for ‘*elt-2*-induced’ genes in animals exposed to the pathogen for a longer duration (24 hours) similarly showed no infection response in *elt-2(RNAi)* animals, suggesting that impaired induction represented a complete failure rather than a delay (S2C Fig).

To identify potential direct ELT-2 targets in the three subsets, we searched gene promoters for the GATA motif core sequence, TGATAA [20,22]. GATA motifs are prevalent in the genome, as targets for various developmental and tissue-specific transcription factors. However, an examination of GATA motif distribution in upstream sequences of *elt-2*-dependent genes revealed a statistically-significant enrichment for GATA motifs in proximal promoter regions, in contrast to a uniform distribution in upstream regions of randomly-selected genes (Fig 1B and S3 Table). Focusing on proximal promoter regions (500 bp) to better differentiate between *elt-2* targets and non-targets, GATA motifs were identified in 72% of the ‘*elt-2* induced’ genes, 50% of ‘*elt-2* regulated’ genes, and 47% of ‘*elt-2* repressed’, compared to 42% in upstream sequences genome-wide, demonstrating a significant enrichment for GATA motifs in promoters of *elt-2*-induced and *elt-2*-regulated genes, but not among ‘*elt-2* repressed’ genes (p = 5.6E-10, 0.004 and 0.1, respectively; hypergeometric distribution). Twelve of the GATA-containing genes were among those tested by qRT-PCR (nine of the ‘*elt-2*-induced’ subset, and three of the ‘*elt-2*-regulated’ subset) and indeed demonstrated *elt-2*-dependent expression (S2 Fig). In addition to enrichment of GATA promoter motifs, 55% of the ‘*elt-2* induced’, and 32% of the ‘*elt-2* regulated’ genes were genes previously reported to be preferentially expressed in the intestine [19,20,31](and Wormbase); only 6/43 (14%) of the ‘*elt-2* repressed’ genes were intestinal, while 12/43 were genes shown to be preferentially-expressed in muscle tissue [32]. Together, these analyses suggest that a large fraction of the *elt-2* regulated genes, in particular of the ‘*elt-2*-induced’ genes, are direct ELT-2 targets. Nevertheless, some ‘noise’ is included in these subsets in the form of genes that are indirectly affected by *elt-2* knock-down. In the case of ‘*elt-2*-repressed’ genes it seems that most are affected indirectly, and probably outside of the intestine, suggesting a negligible contribution of *elt-2* to direct gene repression.

To learn about potential contributions of putative ELT-2 targets to worm physiology, we next examined their associated GO annotations. Among the ‘*elt-2*-regulated’ genes enrichment was found for genes involved in innate immunity and defense responses (represented by 13 genes, p = 8.48E-05, Bonferroni corrected), and for genes with hydrolase activity (37 genes, p = 0.038, not corrected)(S4 Table, highlighted in yellow). The former are genes that were previously shown to respond to infection [22], suggesting that they might have been inappropriately assigned as ‘*elt-2* regulated’ due to a weak response or noisy measurements, and were more likely to be part of the ‘*elt-2* induced’ subset. The more telling members of the ‘*elt-2* regulated’ subset appeared to be the hydrolase genes, which mainly included proteases and lipases, and pointed at regulation of these enzymes as an important function of *elt-2* in adults. Regulation of three of these enzymes by *elt-2* was confirmed by qRT-PCR (S2A Fig). While enrichment for genes annotated as hydrolases is not strictly statistically significant, this may be due to the noise in the ‘*elt-2*-regulated’ list. Supporting the central role of *elt-2* in regulating hydrolytic enzymes in the adult intestine, the overlap between the ‘*elt-2*-regulated‘ gene list and a previously published list of genes specifically expressed in the adult intestine [20] consisted of fifteen genes, four of which are associated with immune defense functions, seven that encode hydrolytic enzymes, and four unknowns (S4 Table).

In embryos, *elt-2* has been shown to contribute significantly to expression of genes encoding structural intestinal proteins [20,33]. However, in agreement with previous results, our microarray data did not reveal effects of *elt-2* knock-down in adults on the expression of *act-5* (microvilli structure), *let-413* (adherens junctions), *eps-8* (apical morphogenesis), and *ifb-2* (intestinal-specific intermediate filament)[22]. In addition, qRT-PCR analysis found no effect of *elt-2* knock-down on the expression of non-hydrolytic genes previously shown to be expressed in the adult intestine: *lmp-1* (lysosomal membrane), *mrp-5* (membranal transport), and *ubl-1* (possibly involved in protein translation)(S3 Fig), whereas hydrolytic enzyme gene expression was reduced in the same RNA samples (as shown in S2A Fig). Together, this indicated that *elt-2* was necessary for specific functions in the adult intestine, but not for all.

ELT-2 was previously shown to function synergistically with ELT-7—a co-expressed intestinal GATA transcription factor—in morphological gut differentiation and in larval gut-specific gene expression [21]. It is possible that redundancy between *elt-2* and *elt-7* masked additional contributions of *elt-2* to intestinal gene expression. Nevertheless, the results presented highlight *elt-2*’s dominant contribution to hydrolytic gene expression.

For the ‘elt-2-induced’ gene subset, all enriched ‘process’ GO annotations were related to defense and innate immune responses (22 genes, p = 1.5E-15)(S4 Table). In addition, ten genes of this subset were annotated with carbohydrate binding, most of which are lectins, which are known to take part in *C*. *elegans* innate immune responses, and have been suggested to play roles in pathogen recognition [34]. These enriched annotations support the dominant role previously proposed for *elt-2* in regulating intestinal innate immune responses. Interestingly, *elt-7* is a member of the ‘*elt-2*-induced’ subset, suggesting participation in immune responses; however, previous work could not identify any significant contribution of *elt-7* to immune protection [22].

### Genetic interactions between *elt-2* and the p38 pathway

**Fig 2:**
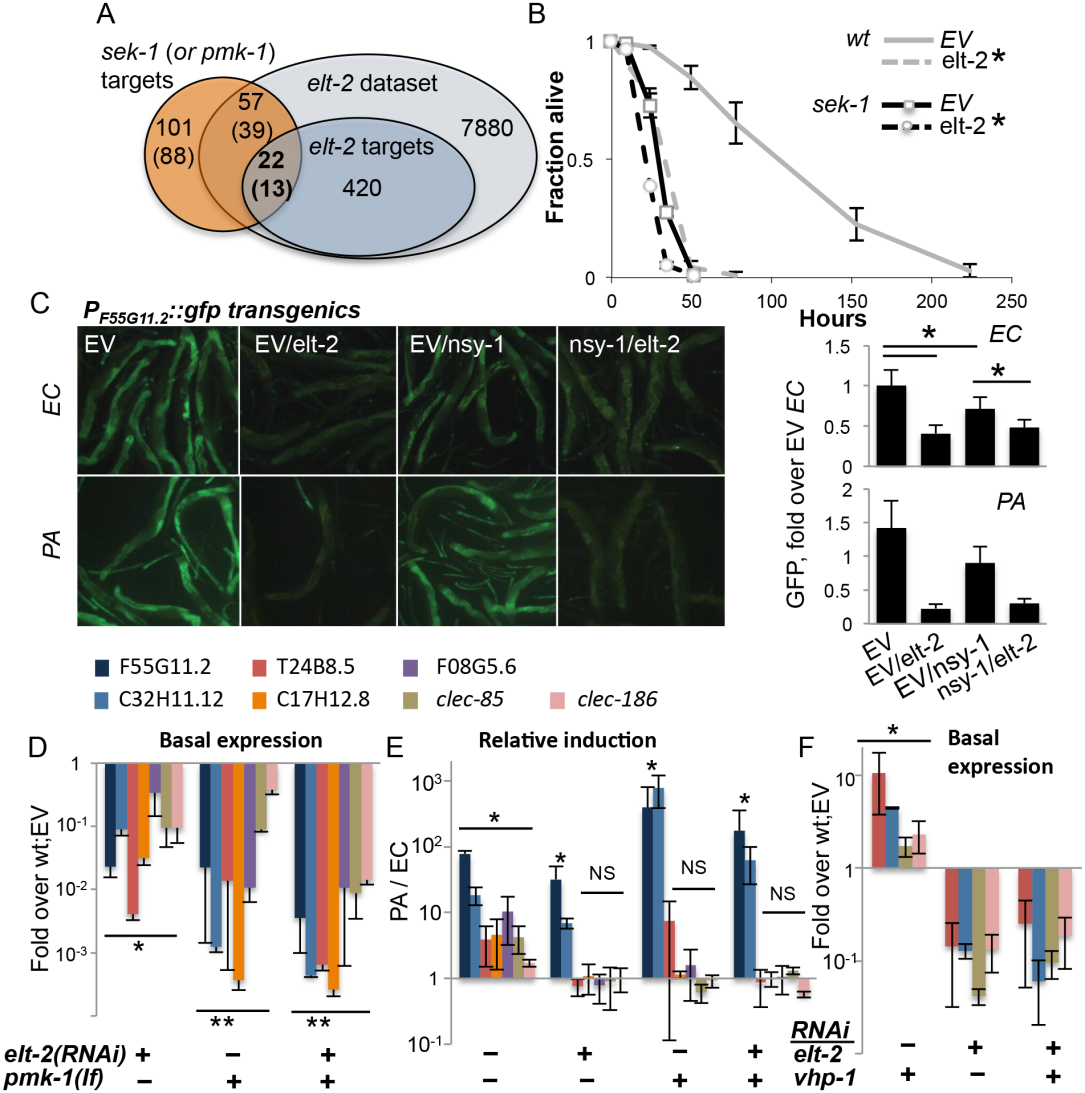
*elt-2* and the p38 pathway co-regulate immune protection. *A*. Overlaps between *sek-1* (or *pmk-1)* targets (Troemel *et al*., 2006), and *elt-2* targets; gene numbers are shown. *B*, Survival curves for wildtype and *sek-1* animals fed with designated RNAi’s during early adulthood, followed by *P*. *aeruginosa* infection; averages ± SDs for three plates (N = 92–151 per group,*p<0.0001 (Logrank test)); shown is a representative of several experiments with similar results. *C*, *P*_*F55G11*.*2*_::*gfp* worms fed with RNAi as designated during adulthood and exposed to *P*. *aeruginosa* (PA, 4 hours, N = 10–19 per group) or *E*. *coli* (EC, N = 19–25); signal quantification shown on the right, *p<3xE-6, ttest. Shown is a representative experiment of two with similar results. *D-F*, Gene expression in wildtype or *pmk-1(km25)* loss-of-function animals fed with designated RNAi’s during larval development. Shown are averages and SDs for two independent experiments *D*,*F*, basal expression (values and statistics relative to values in wt;EV (set to 1 and therefore not shown). *E*, Induction following 12 hours of *P*. *aeruginosa* infection, relative to basal expression in similarly-treated worms grown on *E*. *coli*. *p<0.01, **p<0.0005 (paired t-test); asterisks mark significant differences for individual gene(s), or for each of the genes in a group designated by a line.

ELT-2 acts as a regulator of intestinal development following activation of its expression. This expression is maintained in adults, possibly through autoregulation [35]. ELT-2 was previously shown to be constitutively nuclear [35]. Therefore, to take part in regulation of induced responses (as demonstrated for ‘*elt-2*-induced’ genes) its activity must be modulated by some signal transduction pathway(s). A likely candidate is the p38 pathway, which is known to play an important role in regulating *C*. *elegans* immune responses [4]. Among genes previously described to be regulated downstream to the MAPKK gene *sek-1* or the p38 MAPK gene *pmk-1* [36], and included in our filtered dataset, 38% (22/57) and 33% (13/39), respectively, were also regulated by *elt-2* (p<4E-8)(Fig 2A). This suggested that *elt-2* co-regulated genes with the p38 pathway, potentially downstream to it. To examine this possibility, we knocked down *elt-2* in adult *sek-1(km4)* mutants. While *elt-2* knock-down significantly decreased resistance in wildtype animals, its effect on the already compromised resistance of *sek-1*mutants was marginal (Fig 2B). The fact that overlap between p38 and *elt-2* targets was only partial could reflect technical differences between the two studies, resulting in different coverage of the respective datasets; additionally, it may reflect partially aligned regulatory programs, with some contributions to gene expression that are independent of each other. The survival analysis, showing only marginal exacerbation of infection susceptibility of *sek-1* mutants by elt-2 RNAi is more consistent with the first possibility.

We next turned to gene expression, to further examine the relationship between the *elt-2* and p38 regulatory modules. We began by examining the expression of a GFP reporter controlled by the promoter of F55G11.2, an early immune response gene regulated by both *elt-2* and the p38 pathway [22,36]. RNAi knock-down in adult worms demonstrated that both the p38 MAP3K gene *nsy-1*, and more so *elt-2* were necessary for basal expression from the F55G11.2 promoter (Fig 2C). In response to *P*. *aeruginosa*, F55G11.2 induction was apparent within four hours in control-treated animals, but not in *elt-2* knock-down animals. Disruption of *nsy-1* also reduced immune induction, but not as much as *elt-2* disruption. Similar results were observed in *pmk-1(km25)* mutants, corroborating the co-regulation of F55G11.2 by p38 signaling and *elt-2*, and the dominant contribution of *elt-2* to its expression (S4 Fig).

Using mutants carrying the *pmk-1(km25)* null allele, we expanded our analysis (and increased its sensitivity) by employing qRT-PCR to follow expression of genes potentially co-regulated by p38 signaling and *elt-2*. Because p38-dependent responses are more pronounced in younger worms [37], we measured gene expression at the end of larval development. And while knock-down of *elt-2* during development has more pronounced effects than during adulthood, giving rise to scrawny worms (S1B Fig), *elt-2(RNAi-dev)* worms are healthy enough to reach adulthood and lay eggs. Expression was measured for F55G11.2, and for genes that were part of the overlap between *elt-2* and p38 targets (Fig 2A): C32H11.12 (‘*elt-2*-induced’), T24G8.5, *clec-85* and *clec-186* (all three ‘*elt-2*-regulated’ according to the microarray analysis, and infection-induced in younger animals according to [22]). Two additional p38 targets were included, C17H12.8, and F08G5.6, the latter of which was previously shown to provide protection from infection [22]. All examined genes included proximal-promoter GATA motifs. qRT-PCR demonstrated that the seven genes were all regulated by both *elt-2* and *pmk-1*. Basal expression was significantly reduced following *elt-2* knock-down, compared to age-matched control-treated animals, and was similarly reduced in *pmk-1* mutants (Fig 2D). A twelve-hour exposure to *P*. *aeruginosa* induced the expression of all seven in wildtype animals, but the regulation of this induction divided the genes into two subsets. Induction of 5/7 genes was abolished by either *pmk-1* or *elt-2* disruption, indicating dependence on the two factors. However, F55G11.2 and C32H11.12, which depended on *pmk-1* or *elt-2* for basal expression, were significantly induced above basal levels, even when both *pmk-1* and *elt-2* were disrupted, suggesting that F55G11.2 and C32H11.12 may be regulated by additional factor(s)(Fig 2E). The relative induction observed in these experiments was not apparent in the GFP reporter strain, presumably due to the increased sensitivity of qRT-PCR compared to fluorescence measurements. Similar experiments were performed with adult worms, which showed significantly lower gene induction during infection, but otherwise, similar contributions of *elt-2* and *pmk-1* to gene expression (S5 Fig). Lastly, whether *elt-2* disruption can exacerbate gene repression in *pmk-1* mutants is not clear, since additive effects were observed in two-day adults (S5 Fig), but not in L4 larvae (Fig 2E).

Survival and gene expression analyses in L4 larvae suggested that *elt-2* may be epistatic to *pmk-1*. To examine whether *elt-2* knock-down could abrogate *pmk-1*-dependent gene expression, we knocked down *vhp-1*, which encodes a phosphatase that dephosphorylates and inactivates PMK-1 [10]. Accordingly, knock-down of *vhp-1* caused a significant induction of T24B8.5, C32H11.12, *clec-85* and *clec-186* (Fig 2F). Simultaneous knock-down of *elt-2* abrogated this induction. This was not due to reduced efficiency of vhp-1 RNAi in a double knock-down setting, as *vhp-1* knock-down was able to induce gene expression when mixed with another RNAi (see below). Instead, these results suggested that *elt-2* was essential for *pmk-1* dependent immune gene expression.

### Interactions between *elt-2* and downstream mediators of the p38 pathway

#### ATF-7

**Fig 3:**
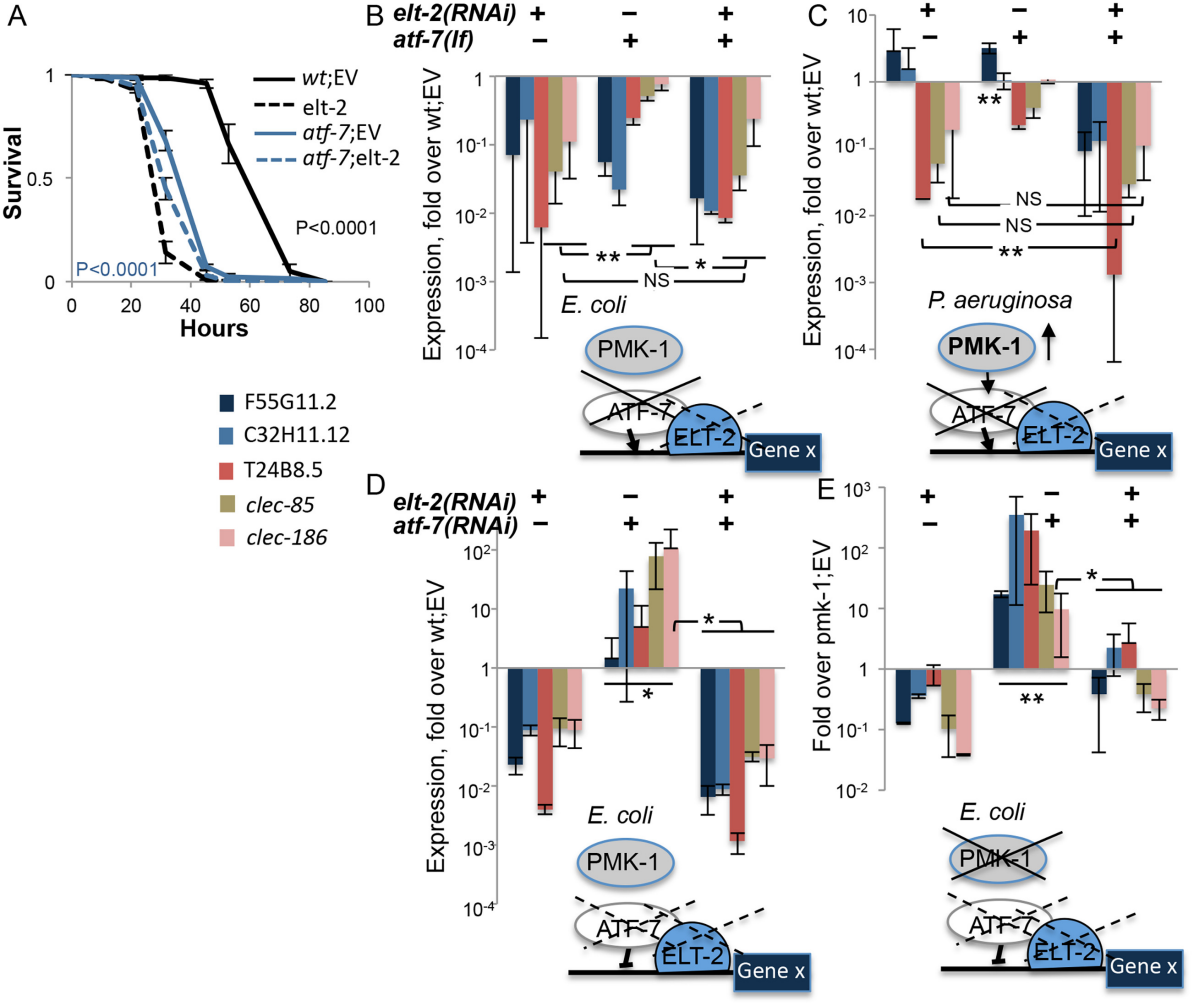
*elt-2* is essential for *atf-7*-dependent immune gene regulation. *A*. Survival curves for wildtype and *atf-7(qd22qd130)* animals fed with designated RNAi’s during development followed by infection. Averages ± SDs for three plates (N = 129–140 per group) in a representative experiment of several others with similar results. *B-E*, Gene expression (log scale) in wildtype, *pmk-1(km25)*, and *atf-7(qd22qd130)* animals, fed with designated RNAi’s during development. Models depict for each panel the mode of disruption, or status, of examined factors (solid-line crosses, loss-of-function mutants; dashed-line crosses, knock-down), placing ELT-2 tentatively at the proximal promoter of immune genes (Gene X) putatively regulated by PMK-1 and ATF-7; *atf-7* is depicted as an activator (arrow) or repressor (blunt-ended arrow), based on disruption effects on gene expression. RNA levels were measured in L4/YA worms. Each panel presents averages and SDs for two independent experiments. Asterisks mark inter-group significance with *p<0.05, **p<0.01, and NS, non-significant (paired t-test) for all genes in the group or in underlined subset.

ATF-7 was reported to regulate gene expression downstream of PMK-1. Normally a repressor of gene expression, its *pmk-1*-dependent phosphorylation during infection transforms it to an activator [11]. Worms carrying the *atf-7(qd22qd130)* loss-of-function allele were reported to be impaired for both gene repression and activation. Given the proposed involvement of *elt-2* in *pmk-1*-dependent immune gene expression, it was of interest to examine how *elt-2* interacted with *atf-7*. Survival analysis showed that *elt-2* knock-down in developing *atf-7* mutants only marginally exacerbated infection susceptibility, as in *pmk-1* mutants (Fig 3A); similar results were observed in worms treated with elt-2 RNAi during adulthood (S6A Fig).

Using *atf-7(qd22qd130)* and *pmk-1(km25)* mutants, in combination with *elt-2* or *atf-7* knock-down, qRT-PCR was employed to examine the involvement of *elt-2* in *pmk-1/atf-7* dependent gene expression. Under normal conditions (growth on *E*. *coli*), both *elt-2(RNAi)* animals and *atf-7* mutants showed a strong reduction in immune gene expression compared to wildtype animals (Fig 3B). Similar results were observed in two-day old adults (S6B Fig). Whereas *atf-7* is expected to function as a repressor under normal conditions, the results suggested that it was necessary (as was *elt-2*) for activating gene expression; this is depicted in the model accompanying Fig 3B. Since *E*. *coli* strain OP50-1 has been previously reported to be weakly pathogenic [38], it is possible that under basal conditions wildtype ATF-7 functions mostly as an activator. While *atf-7* and *elt-2* appeared to regulate the same genes, the relationship between them was not immediately apparent: additive contributions of the two were suggested by expression patterns of F55G11.2 and C32H11.12, but dominance of *elt-2* was suggested by expression patterns of T24B8.5, *clec-85* and *clec-186*, for which *elt-2* knock-down reduced gene expression in wildtype worms or *atf-7* mutants to the same extent with no additive effects.

A similar dichotomy in the relationship between *atf-7* and *elt-2* in regulating target gene expression was observed following infection of wildtype and *atf-7* worms with *P*. *aeruginosa*, which is known to activate PMK-1 (Fig 3C model), and normally induces the expression of all examined genes (Fig 2E). F55G11.2 and C32H11.12 were modestly induced in response to the pathogen even when *elt-2* was knocked down, or in *atf-7* mutants (F55G11.2 only)(Fig 3C). Only a double disruption decreased expression of the two genes to levels below those observed in wildtype animals and abolished induction. This result corroborated the roles of *atf-7* and *elt-2* in positive regulation of immune gene expression, and suggested that for some immune response genes the two factors may provide independent inputs. On the other hand, *clec-85*, *clec-186* and T24B8.5 failed to be induced either in *atf-7* mutants or in *elt-2(RNAi)* animals, and showed in both cases lower RNA levels compared to wildtype control animals, with stronger effects of *elt-2* disruption, and mostly with no additive effects of *atf-7* disruption (with the exception of T24B8.5). This suggested that in the regulation of other immune genes *elt-2* and *atf-7* were epistatic.

While experiments in *atf-7* loss-of-function mutants pointed at roles in gene activation, *atf-7* knock-down experiments in wildtype animals exposed its contributions to gene repression. Knock-down of *atf-7* during larval development resulted in derepression, albeit variable, of all examined genes (Fig 3D). This was abolished by *elt-2* knock-down. Strong derepression was observed only when *atf-7* was knocked down in *pmk-1* mutants, when all ATF-7 molecules are expected to be unphosphorylated and therefore in repressive mode (Fig 3E). Again, *elt-2* knock-down completely abrogated this derepression, supporting the notion that *elt-2* is essential for expression of *atf-7*-regulated genes.

The results presented in Fig 3 demonstrate that *elt-2* is important for *atf-7*-dependent immune gene expression, basal and induced. In particular, gene expression measurements in *pmk-1* mutants suggest that *elt-2* is a master regulator without which *atf-7*-dependent genes cannot be expressed effectively. When ATF-7 was activated, primarily during exposure to *P*. *aeruginosa*, but to a lesser degree also on *E*. *coli*, it co-regulated genes together with *elt-2*, demonstrating additive contributions for some genes, but not for others.

#### SKN-1

**Fig 4:**
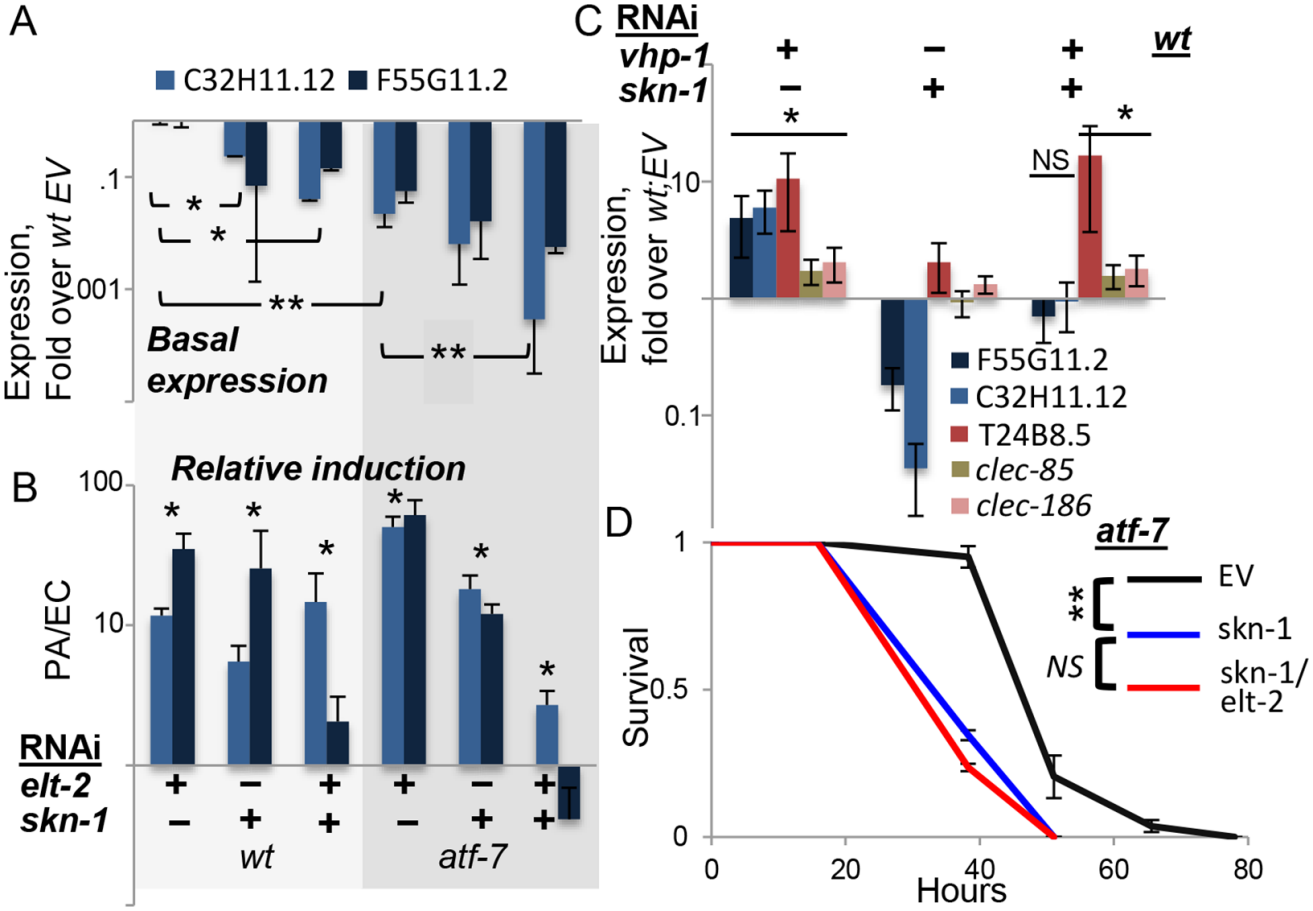
*skn-1* co-regulates gene expression with *atf-7* and *elt-2*. *A-C*, Gene expression (log scale) in wildtype or *atf-7(qd22qd130)* animals fed with designated RNAi’s during development. Averages ± SDs for two independent experiments (or three, in C), with *p<0.05 and **p<0.0005 (t-test) for differences between groups joined by line (A), or between marked groups and their respective references (B). *A*, basal expression following development knock-down, with values and statistics relative to values in wt;EV, not shown. *B*, similar RNAi treatments as in A, followed by exposure to *P*. *aeruginosa* (PA). Responses to PA are shown as fold over basal expression in similarly-treated worms grown on *E*. *coli* (EC). *C*, basal expression following development knock-down; note effective induction of T24B8.5, *clec-85* and *clec-186* by vhp-1 RNAi in the context of a double knock-down. *D*, Survival curves for *atf-7(qd22qd130)* animals fed with designated RNAi’s during development, followed by infection; averages ± SDs for three plates (N = 83–90 per group).

While the expression of *clec-85*, *clec-186* and T24B8.5 were fully explained by contributions from *elt-2* and *atf-7* downstream to the p38 pathway, the expression of F55G11.2 and C32H11.12 was not, and induction, relative to basal expression levels, was still observed when all three were disrupted (Figs 2E and 3C). C32H11.12 was previously shown to be regulated by intestinal SKN-1, and the F55G11.2 promoter is bound by this transcription factor [39,40]. SKN-1 mediates p38-dependent responses to oxidative stress, but was also shown to contribute to immune protection [13–15]. Therefore, we examined whether *skn-1* contributed to the expression of the two genes. Both F55G11.2 and C32H11.12 were repressed when any one of *elt-2*, *skn-1*, or *atf-7* was disrupted (Fig 4A), with accumulating additive effects. However, their infection-induced expression was not significantly reduced until both *skn-1* and *atf-7* were disrupted (Fig 4B). This suggested that each of the three transcription factors contributed to the expression of F55G11.2 and C32H11.12, and that *atf-7* and *skn-1* contributed independently to their induction. Whereas *skn-1* contributed to the expression of these two p38-dependent genes, it did not affect others. Thus, induction of F55G11.2 and C32H11.12 following p38 activation by *vhp-1* knock-down was abolished by *skn-1* knock-down, but induction of T24B8.5, *clec-85* and *clec-186* was not (Fig 4C). Furthermore, not only do *elt-2* and *skn-1* both contribute to F55G11.2 and C32H11.12 expression, but *elt-2* seems to be required for *skn-1*-dependent regulation, as elt-2 RNAi was able to abolish induction of C32H11.12 following *vhp-1* knock-down (Fig 2F).

In summary, *skn-1* seems to be the additional factor needed to explain observed expression patterns of F55G11.2 and C32H11.12. The two genes examined here probably represent a subset of the p38-dependent immune response, regulated not only by *atf-7* and *elt-2*, but also by *skn-1*. Indeed, survival analysis in *atf-7* mutants demonstrated the non-redundant contribution of *skn-1* to infection resistance, and further showed no added contribution of *elt-2*, suggesting that in regulating immune protection *elt-2* works with these two regulators but no additional ones (Fig 4D).

## Discussion

Our expression analyses in *elt-2*-disrupted worms define two dominant roles for *elt-2* in the adult intestine—regulation of hydrolytic, potentially digestive, enzymes, and regulation of defense/immune genes. Whereas *elt-2* has been proposed to regulate all intestinal gene expression, we narrow its role in constitutive intestinal expression by showing that adult *elt-2* is important particularly for expression of genes encoding hydrolytic enzymes, but not those that contribute to intestinal structure. Furthermore, we show for the first time that ELT-2 co-regulates induced immune responses together with ATF-7 and SKN-1, functioning as a tissue-specific master regulator controlling the contribution of the p38 pathway to innate immunity.

### Regulation of immune responses

**Fig 5:**
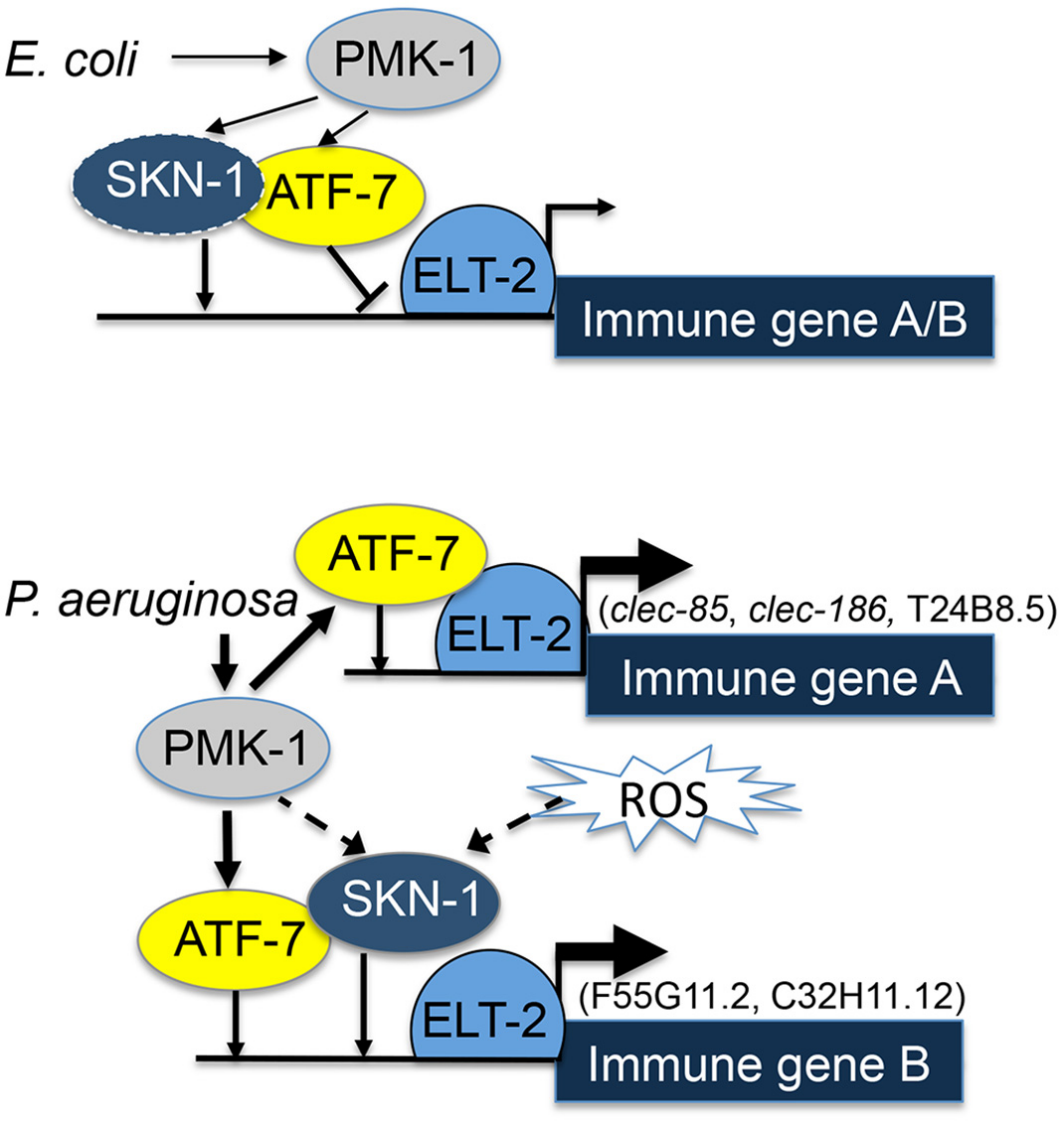
ELT-2-PMK-1-ATF-7-SKN-1 interactions in gene regulation. *A model*. Solid lines represent interactions suggested by results (line thickness is proportional to degree of activation). Dashed lines represent putative alternative options.

ELT-2 was previously shown to be an immune regulator in adult worms, contributing to immune responses and infection resistance [22]. Whereas the vertebrate protein GATA3 activates gene expression following nuclear translocation induced by p38 phosphorylation [41], nuclear localization of the *elt-2* ortholog GATA4 was instead shown to be controlled by the kinase GSK3β [42]. In contrast, ELT-2 was proposed to be constitutively localized to the nucleus [35]. Thus, how *elt-2* contributed to induced responses was not clear, and if p38 was responsible for infection-induced activation of ELT-2, it was still unclear how this was achieved. While our results cannot rule out ELT-2 phosphorylation by the p38 pathway, they suggest a model in which ELT-2 functions as a master regulator of immune gene expression, cooperating with transcription factors activated by the p38 pathway, namely ATF-7 and SKN-1 (Fig 5). Under normal conditions, ATF-7 functions as a repressor and interferes with *elt-2*-dependent gene expression; SKN-1 contributes positively to the expression of some genes (of group B, see Fig 5), but not others (group A). Upon exposure to a pathogen, PMK-1 is activated, phosphorylating ATF-7 and transforming it into a transcriptional activator [11]. In this capacity, ATF-7 cooperates with ELT-2 to induce immune gene expression.

To better fit this model to the results, it is necessary to consider that under normal conditions activated PMK-1 is present (supported by [43,44]); indeed, “normal” conditions include the presence of *E*. *coli* OP50, which is a weak pathogen [38,45]. Thus, by constitutively controlling the interference of ATF-7 with *elt-2*-dependent expression, PMK-1 plays a role in establishing basal levels of immune gene expression.

Whereas co-regulation by ELT-2 and ATF-7 was sufficient to explain immune responses of group A genes, group B genes additionally depended on SKN-1. Our results support a model in which *elt-2* is independently required for *atf-7*- and *skn-1*-dependent gene expression, which could explain the observed additive effects in the contributions of *skn-1* and *atf-7* to the expression of group B genes. SKN-1 can be directly phosphorylated and activated by the p38 pathway [13], but can alternatively be activated by reactive oxygen species (ROS) generated as part of the protective immune response [14]. Furthermore, alternative sources of ROS (e.g. induced by infecting pathogens [46]) may activate SKN-1 independent of the p38 pathway, as suggested by the reported inability of p38 disruption to completely abolish the induction of oxidative stress response genes during infection [14]. A p38-indepedent SKN-1 activation could explain the results presented in Fig 2, demonstrating induction of F55G11.2 and C32H11.12 in infected *pmk-1* mutants. Lastly, a recent report suggested an involvement of the PQM-1 transcription factor in regulating F55G11.2 under normal conditions [47]. *pqm-1* affected F55G11.2 expression, but its contribution appears to be small compared to what we have observed with *elt-2*. While *pqm-1* may provide yet another regulatory input to F55G11.2 gene expression, its contribution is not required for explaining F55G11.2’s expression patterns during infection.

### Regulation of constitutive intestinal gene expression

As a key regulator of intestinal terminal differentiation, the continued expression of *elt-2* in the adult worm has been considered as required for maintenance of intestinal structure and function. Support for this was offered by experiments showing that ectopic *elt-2* expression, or *elt-2* disruption, during embryogenesis, affected expression of intestinal genes, some of which are expressed in adults [20]. However, with only about 10% overlap between adult and embryonic intestinal gene sets it seems that such experiments might reflect *elt-2* contributions in embryos and not necessarily in adults. Differences in *elt-2* contributions in different ages have been described. For example, expression of *ifb-2*, which encodes an intermediate filament protein, is abolished by *elt-2* disruption in embryos, but is unaffected in L1 larvae [21]; similarly, it is unaffected in adults [22](and this study), suggesting diminishing regulatory contributions. It was demonstrated that past embryogenesis, *elt-2* contributed redundantly to intestinal gene expression with a second intestinal GATA transcription factor, ELT-7 [21]: whereas neither disruption of *elt-2*, nor *elt-7*, affected larval *ifb-2* expression, disruption of both abolished this expression; this pattern of redundant regulation was shared by several genes, most of which encode intestinal structural proteins. It is quite possible that *elt-2*, together with *elt-7*, maintains its contributions to expression of structure-related genes in the adult intestine. However, our results suggest a distinct, and dominant, role for *elt-2* in the adult intestine—regulating the expression of hydrolytic enzymes. Such regulation is potentially important for intestinal function (digestion), but also creates a hostile environment for invading pathogens. It is tempting to suggest that the lack of redundancy in regulating these genes (manifested as reduction in gene expression following knock-down of *elt-2* alone) is related to the dominant contribution of *elt-2* for immune responses.

While hydrolytic enzyme genes are the only ones that we found to be enriched among the ‘elt-2-regulated’ genes, they make up only 20% of this subset. It is possible that additional *elt-2*-regulated functions are included in this subset, but are obscured by indirectly regulated genes, which our bioinformatic analysis suggests make up a significant part of this gene subset.

In summary, our genome-wide analysis helps distinguish between basal and pathogen-induced *elt-2-*dependent regulons in the adult worm. Whereas the functional composition of the two appears to be distinct, an overarching theme of anti-bacterial functions is consistent with the idea that bacteria can be both food and pathogens. Additional results further shed light on the largely uncharacterized contribution of *elt-2* to induced responses, revealing cooperation with the transcription factors ATF-7 and SKN-1 downstream to the p38 pathway, and suggesting a function of a tissue-specific master regulator. Whereas *elt-2* contributions to gene expression during and after development seem to differ both compositionally and mechanistically, it seems that its status as a master regulator is maintained in the adult intestine.

## Materials and Methods

### Worm strains

They were obtained from the *Caenorhabditis* Genetics Center and included wild-type N2; *sek-1(km4)*, *pmk-1(km25)* and *atf-7(qd22qd130)* signaling mutants; and *spe-26(it112)* temperature-dependent sterile mutants, which lay unfertilized eggs. *P*_*F55G11*.*2*_::*gfp* worms were designed as described below, and further mated to generated *P*_*F55G11*.*2*_::*gfp;pmk-1(km25)* worms. Bacterial strains included: *E*. *coli* strain OP50-1, *Pseudomonas aeruginosa* strain PA14, and the latter’s GFP-expressing derivative PA14-GFP [48].

### RNAi-mediated knock-down

It was performed with the standard feeding protocol, using bacterial clones from the Ahringer library, with empty RNAi vector (EV) serving as control [22,49]. The exception is atf-7 RNAi, which was from the Open Biosystems library. RNAi feeding was performed for two days, starting at the egg stage (RNAi-dev), or late L4 (RNAi-ad). The protocol used here was previously shown (in worms expressing ELT-2::GFP) to result in a complete knock-down of ELT-2 [22].

### Worm growth and infection

All experiments were carried out using synchronized worm populations grown on *E*. *coli* at 25°C. Infections were performed using the slow killing protocol, typically at 25°C, or when following survival of sensitive strains, at 20°C [48]. Survival analysis of adult *sek-1(km4)* mutants was performed with cdc-25.1(RNAi)-sterilized animals [50], to avoid confounding effects of internal egg hatching. Statistical evaluation of differences between survival curves was performed using Kaplan-Meier analysis followed by the Log-rank test.

### Microarray experiments

Worms were exposed to RNAi (control or elt-2) beginning at the L4 stage, and following two days were transferred either to *E*. *coli* OP50 or to *P*. *aeruginosa* PA14-GFP. Following eighteen hours of exposure (control), or twelve hours (elt-2 RNAi), worms were harvested for RNA extraction and microarray analysis. In a previous study we sought to determine the contribution of colonization (and its associated damage), versus specific pathogen recognition, to differential innate immune responses, and what role *elt-2* played in regulating these responses. Therefore, worms were separated into those that were conspicuously colonized with the GFP-expressing pathogen, and those that were not visibly colonized. Times of exposure to the pathogen were optimized to maximize colonization variability in the population and were therefore shorter in the more susceptible *elt-2(RNAi)* worms. In our previous study we focused on immune responses only in control-treated animals and found them to be identical irrespective of colonization status [51]. In the current analysis we focused on the role of *elt-2* in innate immune responses as a whole, utilizing data from control-treated animals as a reference for comparison. For this purpose, data from colonized and non-colonized worm groups can be pooled into one group—exposure to pathogen. This results in six independent repeats in *control(RNAi)* animals exposed to the pathogen, compared to three repeats of similarly-treated animals exposed to *E*. *coli*; for the *elt-2(RNAi)* animals, the exposure to *E*. *coli* was performed in duplicate, and to the pathogen—in triplicate. RNA was extracted from worms using Trizol (Invitrogen) (100–700 worms per group), and amplified using the MessageAmp II aRNA Amplification Kit (Ambion), labeled with the ULS aRNA Labeling Kit (Kreatech) and co-hybridized to Epoxy (Corning) microarrays spotted with 60-mer oligonucleotides (Washington University Genome Sequencing Center) with a similarly amplified and labeled reference RNA sample [51]. Filtering for high-quality data resulted in 7,880 genes with expression values >2.5 fold over background in >70% of the microarrays. These gene expression profiles were analyzed with the SAM microarray analysis package [52]; a two-class testing configuration was used to identify genes differentially-expressed during infection in untreated worms compared to *elt-2(RNAi)* worms, with a false discovery rate of 9%.

### *P*_*F55G11*.*2*_::*gfp* promoter-reporter strain

A genomic fragment including 1.7 Kb of F55G11.2 upstream region was amplified (annealing: 60°C) using specific primers A-gaagcgcattggtctttga, and B- AGTCGACCTGCAGGCATGCAAGCTttccagcggcggaaact, the latter tailed (capitalized), for subsequent recombinant PCR. This fragment includes part of the F55G11.3 upstream pseudogene, as well as the initial 58 bp of F55G11.2 coding sequence. Recombinant PCR fused this fragment with *gfp*, as previously described, using the nested primer A* (caatttggacacggcaaact) together with the previously described D* primer [53]. Transgenic animals were generated by microinjecting PCR products, together with the *rol-6(su1006)* dominant marker, into worms. Genome integration was subsequently achieved by UV irradiation, as described [54]. GFP signal was quantified in worm images using the MetaMorph analysis software (Molecular Devices).

### Quantitative (q)RT-PCR

RNA extracted as described above was used as template with primers listed in S1 Table. Gene-specific threshold cycle (Ct) values were normalized to the respective actin values, and presented as fold change over normalized values from control-treated animals exposed to *E*. *coli*, or when relative induction was assessed, as fold change in worms exposed to *P*. *aeruginosa* over values in worms of similar genetic background/treatment exposed to *E*. *coli*. Statistical significance was evaluated with a t-test using actin-normalized Ct values.

### Bioinformatics

Management and analysis of gene lists was performed using WormMine (http://www.wormbase.org/tools/wormmine/). Searches for the GATA DNA motif were performed using the MEME suite (http://meme.nbcr.net): FIMO, for analysis of motif distribution; and MAST, for motif prevalence. The DNA motif used for searches was the consensus sequence TGATAA, shared by GATA motifs in different datasets [20,22]. Promoter sequences were retrieved with Worm mart, from Wormbase version WS220. GO analysis was performed with Generic GO Term Finder (http://go.princeton.edu/), using a gene association file downloaded from Wormbase version WS245, and applying Bonferroni correction for p-value calculation (unless otherwise mentioned).

## Supporting Information

S1 Fig *elt-2* knock-down in adult worms and in developing larvae. Knock-down by RNAi feeding (as designated) over two days, starting at L4 (A) or the at egg stage (B). Images taken with identical settings.
(PDF)

S2 Fig qRT-PCR verification of microarray results. RNA levels of designated genes, presented as fold difference over levels in control-treated animals grown on *E*. *coli* (EC) or *P*. *aeruginosa* (PA). RNAi knock-down, as designated was performed during adulthood. *A*. Expression of selected ‘*elt-2*-regulated’ genes (encoding a putative protease, C25B8.3, and two putative lipases, T21H3.1 and Y49E10.16) in wildtype animals; measurements performed in duplicates. *B*. Expression of seven ‘*elt-2*-induced’ genes in wildtype animals exposed to EC or to PA for 12 hours; columns show averages of measurements performed in duplicates, *C*. Expression of three ‘*elt-2*-induced’ genes in *spe-26(it112)* sterile mutants exposed to EC or PA for 24 hours. Averages ± SDs for three independent experiments. Excluding F52H3.7, all of the examined genes contain a proximal promoter GATA motif.
(PDF)

S3 Fig Intestinal genes not regulated by *elt-2* in adults. RNA levels of designated genes in wildtype worms following adult-stage RNAi treatment with designated clones. Shown are averages ± SDs for two independent experiments. NS, non-significant differences.
(PDF)

S4 Fig *elt-2* and *pmk-1* co-regulate F55G11.2 expression. Signal quantification of GFP signal in *P*_*F55G11*.*2*_::*gfp and pmk-1(km25);P*_*F55G11*.*2*_::*gfp* worms fed with RNAi as designated during development, and exposed to *P*. *aeruginosa* (PA, 4 hours, N = 22–35 per group) or *E*. *coli* (EC, N = 25–27); *p<2xE-10, ttest. A comparison to Fig 2C, highlights the stronger induction caused by infection in younger worms. Shown are results for a representative experiment of two showing similar trends.
(PDF)

S5 Fig *elt-2* and the p38 pathway co-regulate immune gene expression in adults. Gene expression in wildtype or *pmk-1(km25)* loss-of-function animals fed with the designated RNAi’s during the first two days of adulthood. Averages and SDs of two experiments, each measured in duplicates. *A*, basal expression (values and statistics are relative to values in wt;EV, not shown). *B*, Induction following 12 hours of *P*. *aeruginosa* infection, relative to basal expression in similarly-treated worms grown on *E*. *coli*. *p<0.05, **p<0.00005 (paired t-test); underlined asterisks mark significance for all four genes.
(PDF)

S6 Fig *elt-2* is essential for *atf-7*-dependent immune gene regulation in adults. *A*. Survival curves for wildtype and *atf-7(qd22qd130)* loss-of-function animals, fed with EV, atf-7 or elt-2 RNAi during adulthood, followed by infection. Shown are averages ± SDs for three plates (N = 129–140 per group) in a representative experiment of several others with similar results. *B*, Gene expression (log scale) in wildtype and *atf-7(qd22qd130)* animals, fed with designated RNAi’s. Basal RNA levels were measured in 2-day old adults. Shown are averages and SDs with *p<0.05 (paired t-test) for two experiments (each measured in duplicates). Asterisks are shown when all genes in the group show statistically-significant differences.
(PDF)

S1 Table Primers used in this study. (XLSX)

S2 Table Raw microarray data. (XLSX)

S3 Table MAST analysis. (XLSX)

S4 Table GO analysis. (XLSX)
