# Supplementary figures and images for: The Developmental Intestinal Regulator ELT-2 Controls p38-Dependent Immune Responses in Adult C. elegans
Source: PLoS Genet. 2015 May 27;11(5):e1005265. doi: 10.1371/journal.pgen.1005265 (PMC4446034; doi:10.1371/journal.pgen.1005265)

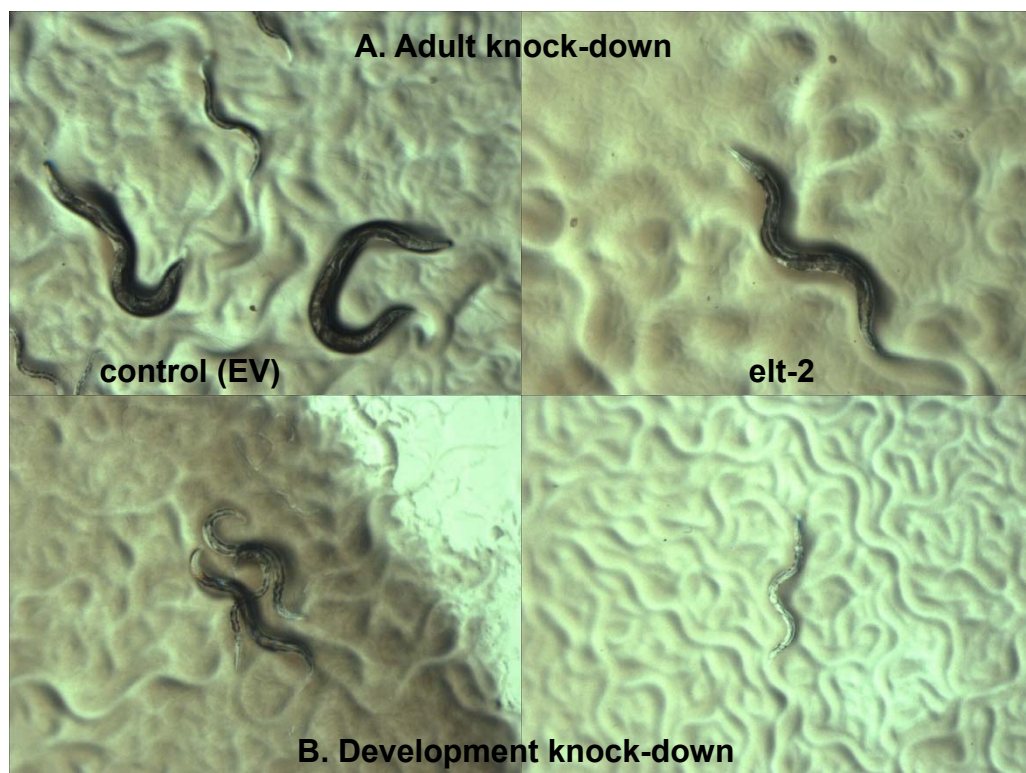

Supplement: S1 Fig — Knock-down by RNAi feeding (as designated) over two days, starting at L4 (A) or the at egg stage (B). Images taken with identical settings. (PDF) [file pgen.1005265.s001.pdf]

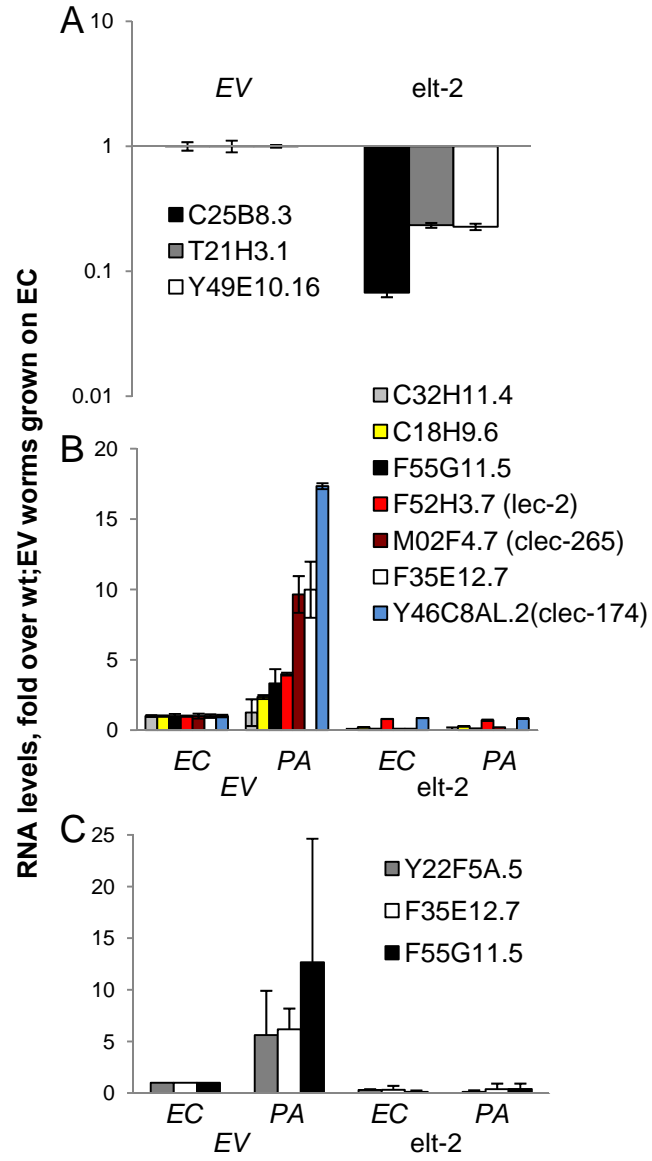

Supplement: S2 Fig — RNA levels of designated genes, presented as fold difference over levels in control-treated animals grown on E. coli (EC) or P. aeruginosa (PA). RNAi knock-down, as designated was performed during adulthood. A. Expression of selected ‘elt-2-regulated’ genes (encoding a putative protease, C25B8.3, and two putative lipases, T21H3.1 and Y49E10.16) in wildtype animals; measurements performed in duplicates. B. Expression of seven ‘elt-2-induced’ genes in wildtype animals exposed to EC or to PA for 12 hours; columns show averages of measurements performed in duplicates, C. Expression of three ‘elt-2-induced’ genes in spe-26(it112) sterile mutants exposed to EC or PA for 24 hours. Averages ± SDs for three independent experiments. Excluding F52H3.7, all of the examined genes contain a proximal promoter GATA motif. (PDF) [file pgen.1005265.s002.pdf]

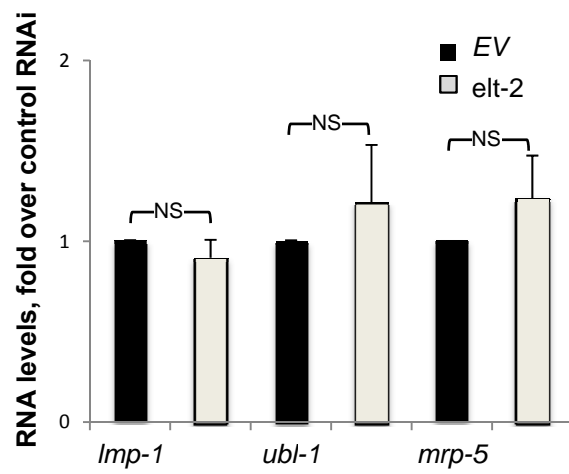

Supplement: S3 Fig — RNA levels of designated genes in wildtype worms following adult-stage RNAi treatment with designated clones. Shown are averages ± SDs for two independent experiments. NS, non-significant differences. (PDF) [file pgen.1005265.s003.pdf]

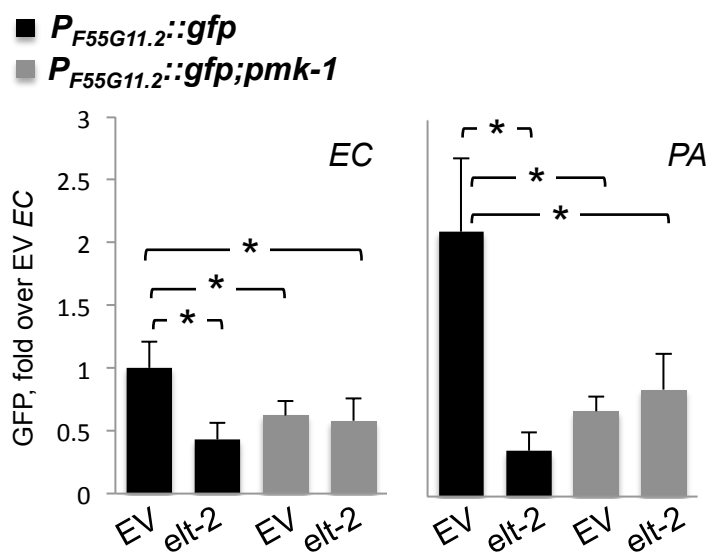

Supplement: S4 Fig — Signal quantification of GFP signal in P F55G11.2::gfp and pmk-1(km25);P F55G11.2::gfp worms fed with RNAi as designated during development, and exposed to P. aeruginosa (PA, 4 hours, N = 22–35 per group) or E. coli (EC, N = 25–27); *p<2xE-10, ttest. A comparison to Fig 2C, highlights the stronger induction caused by infection in younger worms. Shown are results for a representative experiment of two showing similar trends. (PDF) [file pgen.1005265.s004.pdf]

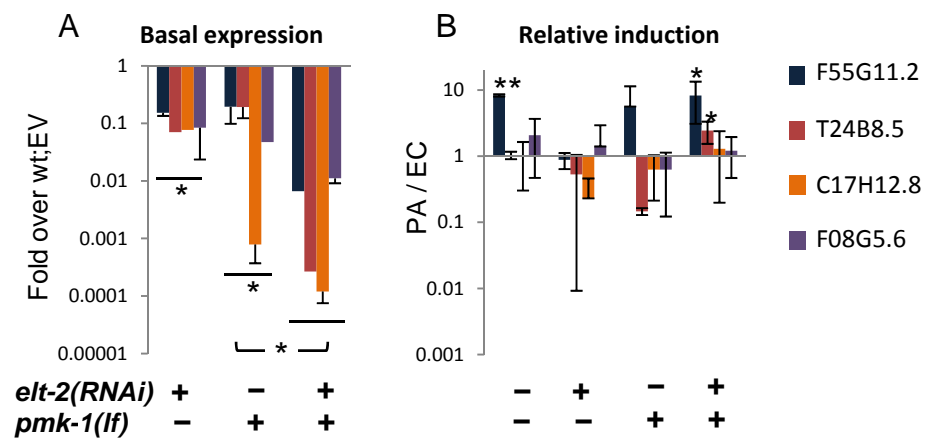

Supplement: S5 Fig — Gene expression in wildtype or pmk-1(km25) loss-of-function animals fed with the designated RNAi’s during the first two days of adulthood. Averages and SDs of two experiments, each measured in duplicates. A, basal expression (values and statistics are relative to values in wt;EV, not shown). B, Induction following 12 hours of P. aeruginosa infection, relative to basal expression in similarly-treated worms grown on E. coli. *p<0.05, **p<0.00005 (paired t-test); underlined asterisks mark significance for all four genes. (PDF) [file pgen.1005265.s005.pdf]

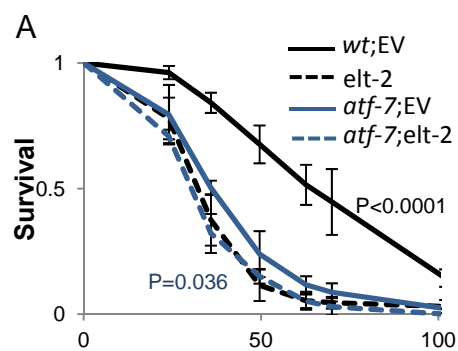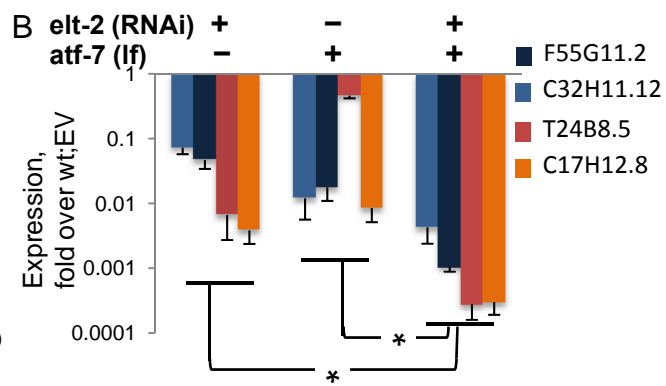

Supplement: S6 Fig — A. Survival curves for wildtype and atf-7(qd22qd130) loss-of-function animals, fed with EV, atf-7 or elt-2 RNAi during adulthood, followed by infection. Shown are averages ± SDs for three plates (N = 129–140 per group) in a representative experiment of several others with similar results. B, Gene expression (log scale) in wildtype and atf-7(qd22qd130) animals, fed with designated RNAi’s. Basal RNA levels were measured in 2-day old adults. Shown are averages and SDs with *p<0.05 (paired t-test) for two experiments (each measured in duplicates). Asterisks are shown when all genes in the group show statistically-significant differences. (PDF) [file pgen.1005265.s006.pdf]
